# Obesity, Overweight, and Perceptions about Body Weight among Middle-Aged Adults in Dar es Salaam, Tanzania

**DOI:** 10.5402/2012/368520

**Published:** 2012-08-28

**Authors:** Alfa J. Muhihi, Marina A. Njelekela, Rose Mpembeni, Ramadhani S. Mwiru, Nuru Mligiliche, Jacob Mtabaji

**Affiliations:** ^1^Intervention Thematic Group, Ifakara Health Institute, P.O. Box 53, Ifakara, Morogoro, Tanzania; ^2^Department of Physiology, Muhimbili University of Health and Allied Sciences, Dar es Salaam, Tanzania; ^3^Department of Epidemiology and Biostatistics, Muhimbili University of Health and Allied Sciences, Dar es Salaam, Tanzania; ^4^Department of Nutrition, Harvard School of Public Health, Boston, MA 02115, USA; ^5^Department of Anatomy and Physiology, Weill Cornell Medical College in Qatar, Qatar Campus, Doha, Qatar; ^6^Department of Physiology, Catholic University of Health and Allied Sciences-Bugando, Mwanza, Tanzania

## Abstract

*Background*. Prevalence of obesity is increasing throughout the world at an alarming rate. Appropriate perception of one's own body weight is important for improved weight control behavior. This study was conducted to determine the prevalence of overweight and obesity and assess perception of body weight among middle aged adults in Dar es Salaam, Tanzania. *Methods*. Structured questionnaire was used to collect sociodemographic and lifestyle information including perception about body weight. Anthropometric measurements were taken by a trained person following standard procedures. *Results*. Prevalence of obesity was 13% and 36% among men and women, respectively. There was significant gender difference in perception of body weight (12% and 25% of men and women perceived their body weight as overweight). Only 2% of women perceived themselves as obese whereas none of the men did so. Among overweight men, only 22% perceived themselves as overweight/obese compared to 38% of overweight women who perceived themselves as overweight/obese. Overall, majority of the participants (87%) were willing to lose weight. *Conclusions*. There is a great difference between perceived and actual body weight with men underestimating their body weight more than women. Educational programs regarding overweight and obesity and the associated health consequences are highly recommended in Tanzania.

## 1. Introduction

The prevalence of obesity is increasing throughout the world at an alarming rate. According to World Health Organization (WHO), the burden of obesity has doubled in the past two decades and that by the end of 2008, there were 1.5 billion overweight adults aged 20 years and above. Over 200 million men and nearly 300 million women were obese [1]. Between 1980 and 2008, the mean body mass index (BMI) increased at a rate of 0.4 kg/m^2^ per decade worldwide [2]. Over a period of 10 years, the prevalence of obesity was reported to increase from 2.3% to 19.6% in several developing counties [3]. In Tanzania, obesity has been shown to be higher among urban men and women compared to their rural counterparts [4]. The recent estimates show that the prevalence of obesity is 13% and 36% among urban men and women, respectively [5]. 

Several factors contributing to increasing prevalence of obesity have been discussed [6–14]. However, urbanization and globalization of food production and marketing are the two most important factors fueling the rise in prevalence of obesity in developing countries [8]. Other factors like hereditary [6, 9, 11], sedentary lifestyle [7, 10, 14], and sociocultural factors [13] should also be appreciated as contributors to increasing obesity in these countries. 

The perception about body weight is influenced by several factors including culture and ethnicity [15, 16]. In developed countries, a thin body is an ideal and preferred among females [17]. In developing countries, heavier body is the preferred one although there is a shift towards a thin body among people of higher class [18]. In additional to sociocultural factors, HIV pandemic has had a great impact on people's perception about body weight in Tanzania [19]. Many people prefer to be overweight or obese so as not to look suspicious of having HIV/AIDS. 

The findings from the first International Body Project (IBP-I), which was conducted across 10 major geographical regions of the world showed significant cross-regional differences in the perception about an ideal female body, with heavier body preferred in low compared to high socioeconomic sites. Another study conducted in Seychelles showed that appropriate perception of one's body weight as too high was associated with socioeconomic status indicators, female sex, and being actually overweight [20]. Women have been reported to have higher false perception of their body weight compared to men [21]. Other numerous surveys have also shown that women are generally dissatisfied with their bodies in comparison to men [22, 23]. 

Despite an increasing prevalence of obesity in Dar es Salaam, no studies have examined people's perception of body weight in this population. Because an appropriate perception of one's own weight is important for improved weight control behavior [24], understanding people's perception of obesity will help in designing educational programs to address obesity and other related chronic diseases in Tanzania. We assessed the perception of body weight among middle aged adults in the business capital of Dar es Salaam, Tanzania. 

## 2. Methods

### 2.1. Study Design, Population, and Sampling Methods

The design of the study was cross-sectional using quantitative methods. The study was conducted in Temeke district in Dar es Salaam region. Temeke district has a total of 24 wards with an estimated population of 768,451. Majority of them (94%) reside in urban areas [25]. A sampling frame of all wards was obtained from the Temeke district authorities and five wards were randomly selected. The selected wards were Mbagala, Mji mwema, Kigamboni, Kimbiji, and Vituka. From each selected ward, a sampling frame of streets from the ward executive officer was used to randomly select one street. A list of all men and women aged 44–66 years residing in the selected street was prepared by the street leaders. A total of 250 participants (50 from each street) were randomly selected and invited to participate in the study. Two hundred and nine participants were enrolled into the study. The refusal rate was 16.4% mainly because of fear of HIV testing from blood samples that were collected for analysis of lipid profile. 

### 2.2. Assessment of Socioeconomic Status

Socioeconomic status was evaluated by using a structured questionnaire. Participants were grouped into three groups according to their total monthly income in Tanzanian shillings (Tshs): low income (≤50,000/=), medium (51,000–120,000/=), and high income (>120,000/=). As for formal education, participants were categorized as: no formal education (could not read, write, or solve simple mathematics), primary education (attended primary school up to 7 years), secondary education (included those with ordinary and advanced secondary education as well as vocational training), and college and university level education. 

### 2.3. Anthropometric Measurements

Data collection procedures for this study have been described elsewhere [5]. Anthropometric measurements were conducted by a trained physician and one study nurse. Body weight was measured with subject standing and wearing light clothes and without shoes to the nearest 0.1 kg using a digital scale (Tanita, Tokyo, Japan). Height was measured to the nearest 0.5 cm using a portable stadiometer. BMI was then calculated as weight in kilograms divided by height squared in meters (kg/m^2^), and categorized as underweight (BMI < 18.5 kg/m^2^), normal (BMI 18.5–24.9 kg/m^2^), overweight (BMI 25.0–29.9 kg/m^2^), and obese (BMI ≥ 30.0 kg/m^2^) [26]. Hip circumference (HC) and waist circumference (WC) were measured to the nearest 0.5 cm using a flexible tape measure. Abdominal obesity was defined as WC ≥ 102 cm among men and ≥88 cm among women. Waist-to-hip ratio (WHR) was calculated by dividing WC by HC, and high WHR was defined as ≥0.9 among men and ≥0.85 among women.

### 2.4. Perception about Body Weight

The perception about body weight in this population of middle-aged men and women was assessed using the following question.

1. For the past one year, has your body weight increased, decreased, or stayed the same?
2. In the past one year, have you seriously tried to lose weight? (Yes, No).
3. How do you perceive your current body weight? (Underweight, normal for my age, overweight/obese).
4. Would you like/wish to lose weight? (Yes, No).

In addition, sociodemographic information, occupation, smoking habits, alcohol intake, physical activity, and general health status were assessed using structured questionnaires that were administered by two trained research assistants. To assess the socioeconomic status we asked about the monthly income of the respondents. Participants' monthly income in Tanzanian shillings (Tshs) was categorized into three levels: less than 50,000 Tshs, 50,000 to ≤80,000 Tshs, and more than 80,000 Tshs (1,500 Tshs = 1 US$). Level of education was categorized into four levels as no formal education, primary education (≤7 years of schooling), secondary education, and college/university level. Current smoking and alcohol drinking statuses were also evaluated. The Sub-Saharan Africa Activity Questionnaire (SSAAQ) was used to assess physical activity [27]. We used the updated compendium of physical activity [28] to code the metabolic equivalent (MET) intensity levels of the various physical activities. 

### 2.5. Data Analysis

Statistical analysis software (SAS 9.2, Institute Inc., North Carolina, USA) was used for data entry and analysis. The general characteristics of the study population were described using standard descriptive statistics means, standard deviations, and frequencies. Difference in the prevalence of overweight and obesity by gender was assessed using chi-square (*χ* ^2^) test. In all the analyses, *P* ≤ 0.05 was considered statistically significant. The study was approved by ethical review committee of the Muhimbili University of Health and Allied Health Sciences (MUHAS) and an informed consent was obtained from all participants prior to the study.

## 3. Results

### 3.1. General Characteristics of the Study Population

The general characteristics of the study population are summarized in Table 1. The mean age and BMI of the participants were 53.6 ± 6.2 years and 27.4 ± 14.6 kg/m^2^, respectively. The mean age was higher for men (*P* = 0.01) while mean BMI was higher for women (*P* < 0.01). Statistically significant gender differences were also observed for education, occupation, monthly income, smoking, and alcohol drinking status. As demonstrated by energy expenditure in MET hours/day, women tended to be more physically active than men.

### 3.2. Current Body Weight 

As presented in Table 1, the overall prevalence of overweight and obesity in this population of young and middle-aged adults as determined by BMI was 32.54% and 23.44%, respectively. The prevalence of both overweight and obesity was higher among women compared to men (31% versus 34% for overweight and 36% versus 13% for obesity). This gender difference in the prevalence of overweight and obesity was statistically significant (*P* < 0.001). 

### 3.3. Perceived Changes in Body Weight

Overall, half (50%) of the participants perceived that their body weight had not changed in the past one year (46% men and 55% women). More than one-third (34%) of the participants reported that their body weight had increased in the past one year. However, there were no gender differences in perception about weight change. Only 47 participants (23 men and 24 women had tried to seriously lose their weight (Table 2).

### 3.4. Perception about Current Body Weight

Forty participants (19%) perceived their body weight as overweight or obese. Among female participants, 24 (26%) and 2 (2%) perceived themselves as overweight and obese, respectively. As for male participants, only 14 (12%) considered themselves as overweight and none perceived his weight as obese. These differences in perception about body weight among men and women in this study were statistically significant (Table 2). 

Among overweight and obese men (*n* = 54), only 12 (22%) correctly estimated their weight as overweight or obese while the majority (71%) perceived themselves as normal weight and 7% as underweight. For the overweight and obese women (*n* = 63), 24 (38%) correctly estimated their weight as overweight or obese. Nearly half (49%) estimated their weight as normal weight and 13% as underweight. Generally, overweight and obese men tended to underestimate their body weight more than women (78% versus 62%). The difference between perceived and actual body weight as indicated by BMI was statistically significant in both men and women (*P* = 0.001 and *P *= 0.003, resp.) (Table 3).

## 4. Discussion

We found statistically significant gender differences in the prevalence of obesity in this population of middle-aged men and women. There were also differences in the perceived and actual body weights among men and women. Overall, overweight and obese men tended to underestimate their body weight more than women. These findings concur with those reported from studies conducted in other African countries [20, 29], Asia [30], Europe [31] and in the United states [32]. However, a study conducted in a high Atlas Moroccan population found that women underestimated their overweight more than men [21].

Obesity constitutes a major public health problem and increases the risk for cardiovascular diseases, which are the main causes of mortality globally [33]. Our findings indicate that the prevalence of obesity is higher among women compared to men (36% versus 13%). A previous study conducted in Dar es Salaam and two other rural areas indicated a higher prevalence of obesity among urban residents of Dar es Salaam compared to their counterpart rural dwellers in both men and women [4]. A higher prevalence of obesity among women has also been noted in other population surveys [34–36]. 

Despite a higher prevalence of obesity in this urban population, there is a tendency towards underestimation of body weight. Majority (78%) of the overweight and obese men did not perceive themselves as overweight or obese, and are therefore likely to gain more weight, because an appropriate perception of person's own weight is favorable to improved weight control behavior [24]. In Cameroon, it was reported that men aged 40 to 60 years underestimated their weight and wished to gain more weight compared to other age categories while among women, the difference in the under-estimation of weight was not statistically significant [21]. In another study in Seychelles among persons with excess weight, 63.5% of overweight males, 45.1% of overweight females, 23.6% of obese males, and 17.2% of obese females did not perceive their weight as being too high [20]. Compared to men, overweight and obese women in our study were also less likely to underestimate their weight.

Cultural factors have been shown to influence perception about body weight and consequently prevalence of obesity [37, 38]. Dar es Salaam is a multicultural city with its inhabitants coming from all corners of Tanzania. As a result, residents of Dar es Salaam do not have a distinct culture and have adopted an urban lifestyle. There is therefore a need to explore whether cultural factors play significant role on perception about body weight and the increasing prevalence of obesity in this urban setting of Dar es Salaam. 

Despite its cross-sectional design which does not allow drawing inference on the direction of the association, the strengths of our study include its population-based design and the use of actually measured weights and heights. Other studies have assessed perception about body weight by relying on self-reported values of weights and heights, which can also be over- or under-estimated. 

## 5. Conclusions

It is evident from our findings that majority of overweight and obese individuals do not consider themselves as having excess weight. Health education and other interventions are needed to address issues related to perception about body weight. Health professionals should take the lead in informing and making people realize the health risks associated with excess body weight. Adoption of healthy lifestyles such as good diet and engaging in physical exercises should be encouraged in this population of middle aged adults in Dar es Salaam. Some false beliefs that an overweight or obese person is healthier should be addressed as well. To broadly understand issues surrounding perception about body weight in Tanzania, a large study covering both urban and rural settings is recommended. 

## Figures and Tables

**Table 1:** General characteristics of the study population.

Characteristic	Men (*N* = 115)		Women (*N* = 94)		*P* value
	Mean ± Sd	*n* (%)	Mean ± Sd	*n* (%)	
Age (years)	54.6 ± 6.1		52.5 ± 6.1		0.010
BMI(kg/m^2^)	26.6 ± 6.4		28.3 ± 6.5		0.0004
Ward of residence					0.43
Mbagala		21 (18)		16 (17)	
Mjimwema		11 (10)		15 (16)	
Kimbiji		24 (21)		12 (13)	
Kigamboni		30 (26)		27 (29)	
Vituka		29 (25)		24 (25)	
Married					<0.001
Yes		98 (85)		54 (57)	
No		17 (15)		40 (43)	
Education					0.011
No formal education		10 (9)		21 (22)	
Primary education		7 (6)		8 (9)	
Secondary education		64 (56)		50 (53)	
College or university		34 (30)		15 (16)	
Occupation					<0.001
Not employed		9 (8)		29 (31)	
Public/private Institution		16 (14)		11 (12)	
Self-employed/Business		41 (36)		34 (36)	
Farmers		49 (42)		20 (21)	
Monthly income (Tshs)					0.004
Low (<50,000)		65 (56)		67 (72)	
Medium (50,000–80,000)		26 (23)		6 (6)	
High (>80,000)		24 (21)		21 (22)	
BMI category (kg/m^2^)					<0.001
Underweight		7 (6)		3 (3)	
Normal weight		54 (47)		28 (30)	
Overweight		39 (34)		29 (31)	
Obese		15 (13)		34 (36)	
Current smoker					<0.001
Yes		26 (23)		2 (2)	
No		89 (77)		92 (98)	
Currently drink alcohol					0.003
Yes		42 (37)		17 (18)	
No		73 (63)		77 (82)	
Physical activity (MET hours/day)					0.060
<26		39 (34)		31 (33)	
26–37		45 (39)		24 (25)	
>37		31 (27)		39 (42)	

**Table 2:** Weight change and perception about body weight among men and women.

	All (*N* = 209)	Men (*N* = 115)	Women (*N* = 94)	*P* value
	*n* (%)	*n* (%)	*n* (%)	
Weight change in the past 1 year				0.40
Has decreased	33 (16)	19 (17)	14 (15)	
Has not changed	105 (50)	53 (46)	52 (55)	
Has increased	71 (34)	43 (37)	28 (30)	
Ever tried to lose weight				0.41
Yes	47 (22)	23 (20)	24 (26)	
No	162 (78)	92 (80)	70 (74)	
Perception about current weight				0.01
Underweight	39 (19)	20 (18)	19 (20)	
Normal weight	130 (62)	81 (70)	49 (52)	
Overweight	38 (18)	14 (12)	24 (26)	
Obese	2 (1)	0 (0)	2 (2)	

**Table 3:** Perceived body weight in comparison to the actual body weight as indicated by BMI.

Body mass index (BMI)	Perceived body weight			*P* value
	Underweight	Normal weight	Overweight/Obese	
Men				0.001
Underweight	4 (57%)	3 (43%)	0 (0%)	
Normal weight	12 (22%)	40 (74%)	2 (4%)	
Overweight or obese	4 (7%)	38 (71%)	12 (22)	
Women				0.003
Underweight	1 (33%)	2 (67%)	0 (0%)	
Normal weight	10 (36%)	16 (57%)	2 (7%)	
Overweight or obese	8 (13%)	31 (49%)	24 (38%)	

## Appendix

For more details see Tables 1, 2, and 3.
